# Notes on braconid wasps (Hymenoptera, Braconidae) parasitising on *Agrilus mali* Matsumura (Coleoptera, Buprestidae) in China

**DOI:** 10.3897/zookeys.867.36170

**Published:** 2019-07-30

**Authors:** Liang Ming Cao, Yan Long Zhang, Cornelis van Achterberg, Zhi Yong Wang, Xiao Yi Wang, Wen Xia Zhao, Zhong Qi Yang

**Affiliations:** 1 The Key Laboratory of Forest Protection, State Forestry and Grassland Administration of China, Research Institute of Forest Ecology, Environment and Protection, Chinese Academy of Forestry, Beijing 100091, China Research Institute of Forest Ecology, Environment and Protection, Chinese Academy of Forestry Beijing China; 2 Institute of Insect Sciences, Zhejiang University, Hangzhou 310058, China Zhejiang University Hangzhou China; 3 Leshan Normal University, Leshan, 614000, China Leshan Normal University Leshan China

**Keywords:** *
Agrilus mali
*, Braconidae, China, new record, parasitoid wasps, *
Malus sieversii
*

## Abstract

Braconid parasitoids reared from *Malus sieversii* and *Malus domestica* trees in NW China infested by *Agrilus mali* Matsumura (Coleoptera, Buprestidae) are illustrated and discussed. Six species were found parasitising *Agrilus mali* in NW China, namely, *Atanycolus ivanowi* (Kokujev) (Braconinae), *Doryctes undulatus* (Ratzeburg), *Pareucorystes varinervis* Tobias, *Polystenus rugosus* Foerster, *Spathius sinicus* Chao, and *Spathius brevicaudis* Ratzeburg (Doryctinae). All listed species are newly recorded parasitoids of *Agrilus mali*. *Pareucorystes varinervis* and *Spathius brevicaudis* are new records for the Chinese fauna, but *Spathius brevicaudis* has been recorded from Taiwan before. Both sexes of *Spathius brevicaudis* are redescribed here to allow inclusion in the recent revision of the Chinese *Spathius* species. An identification key to the six braconid parasitoids of *Agrilus mali* in NW China is provided.

## Introduction

**Figure 1.**
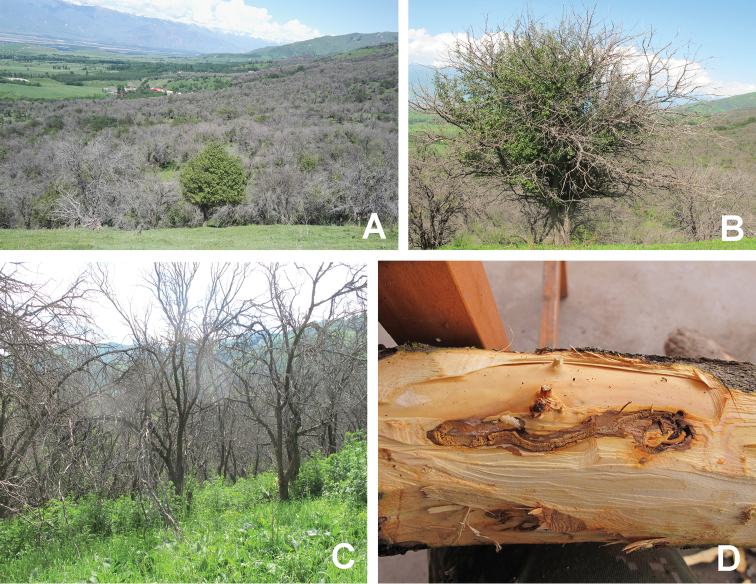
**A** Trees of *Malus sieversii* damaged by *A. mali***B** A single tree of *Malus sieversii* damaged by *A. mali***C** Dead branches because of *A. mali***D** Trunk of *Malus sieversii* damaged by *A. mali*.

The apple buprestid, *Agrilus mali* (Coleoptera: Buprestidae), is considered to be a dangerous pest in China of apple trees. Recently, a large area of wild *Malus sieversii* has been killed by *Valsa mali* Miyabe et Yamada and *Cytospora mandshurica* Miura, after infection by *A. mali* (Figure 1A, B, C). Its larvae feed under the bark on the phloem, which weakens the nutrient transportation and results in depressed and dark-coloured dead bark over the affected areas (Figure 1D). Heavy infestations result in dead branches and, eventually, in the death of the whole tree (Figure 1D). Besides apple trees, also other fruit trees (e.g., crab-apple, pear, peach, and cherry) are attacked.

**Figure 2.**
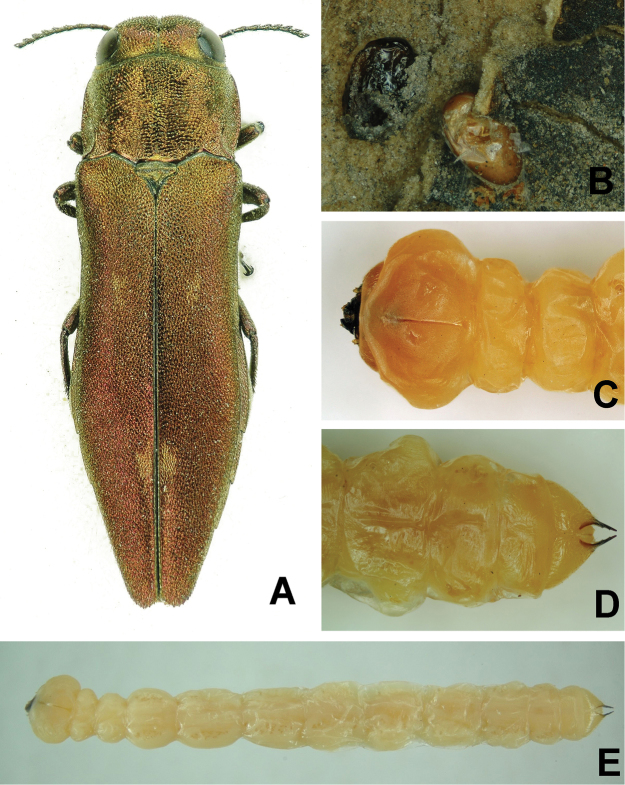
**A** Adult of *A. mali***B** Eggs of *A. mali***C** Head and thorax of larva, dorsal view **D** Abdominal tip of larva, ventral view **E** Larva of *A. mali*, ventral view.

Adult, egg and larval stages of *Agrilus mali* are shown in Figure 2; *A. mali* is widely distributed in Asian Russia, Japan, the Korean peninsula, and north China, and is a common species in apple orchards. In 1995 it was reported attacking the endangered wild apple (*Malus sieversii*) in Xinyuan County, Ili Kazakh Autonomous Prefecture, Xinjiang Province (Wang et al. 1995). This tertiary relict is the sole ancestor of most cultivars of the domesticated apple, *Malus domestica*. With its high genetic diversity, the forest is seen as the most significant national gene pool of apple, even to the world. Recently, *A. mali* became the major pest of *M. sieversii* and 48.6% of the Xinjiang wild apple forest was damaged, over 4866.67 m^2^, and in some areas like Xinyuan County and Gongliu City, most trees died.

Using organic insecticides has allowed the control of *A. mali* in orchards, but in the wild this is ineffective as the trees are scattered over a vast area. The damage of *A. mali* is erratic, wide-spread, and frequent spraying of chemicals will be another threat to the vulnerable local ecosystem. Therefore, biological control is considered as the best countermeasure and a survey about the natural enemies of *A. mali* has been carried out in recent years.

## Materials and methods

This study is based on specimens retained in the Entomological Museum of Chinese Academy of Forestry (Beijing, China) and the Naturalis Biodiversity Center (Leiden, the Netherlands). Natural enemy surveys of *A. mali* were conducted in Xinjiang, Shaanxi and Qinghai Provinces from 2011 to 2018. Trunk bark of stressed trees was peeled off to search for *A. mali* larvae and associated parasitoids. The larvae and possible parasitoid cocoons were placed singly in vials (12 mm in diameter and 75 mm in length), each containing a piece of filter paper dipped in distilled water for moisture. The vials were plugged tightly with sterilised cotton and maintained at 22–25 ℃ in the rearing room. Parasitoid cocoons were successively reared to adults. Specimens were examined with a Nikon SZH 1500 stereomicroscope. Photographs were taken with an Olympus CX31 microscope with the UV–C Optical Totally Focuses System developed by Beijing United Vision Technology Co., Ltd. Terminology and measurements follow van Achterberg (1993).

## Taxonomy

### Braconinae

#### Atanycolus ivanowi

Taxon classificationAnimaliaHymenopteraBraconidae

(Kokujev, 1898)

7fb0b03a-6f4d-5dd9-833d-46834f02aeba

**Figure 3.**
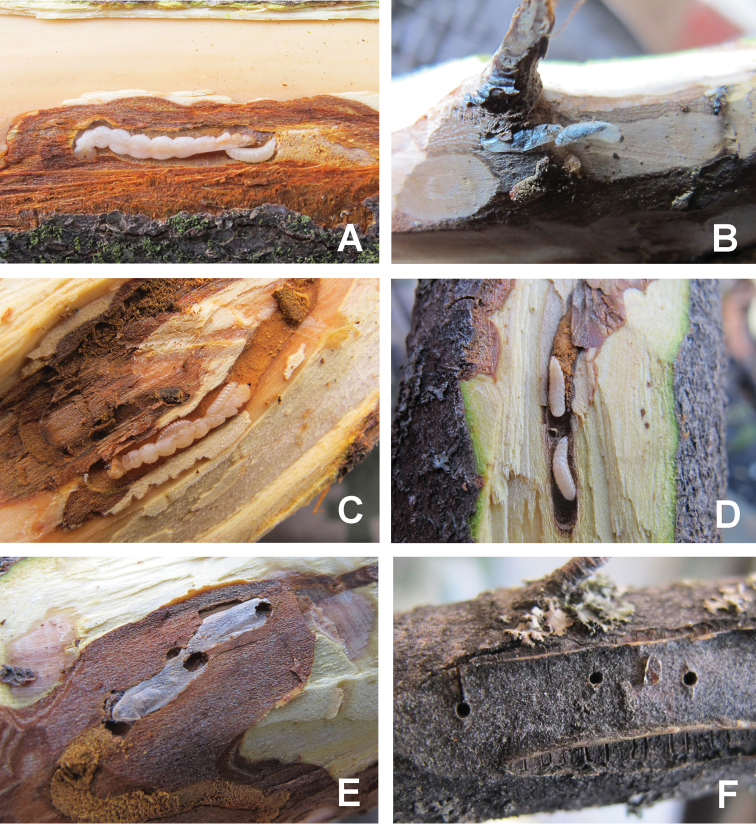
**A** Larva of *Atanycolus ivanowi* on larva of *A. mali***B** Cocoons of *Atanycolus ivanowi***C** Third instar larvae of *Doryctes undulatus***D** Fourth instar larvae of *Doryctes undulatus***E** Empty cocoons of *Doryctes undulatus***F** Emergence hole of *Doryctes undulatus*.

**Figure 4.**
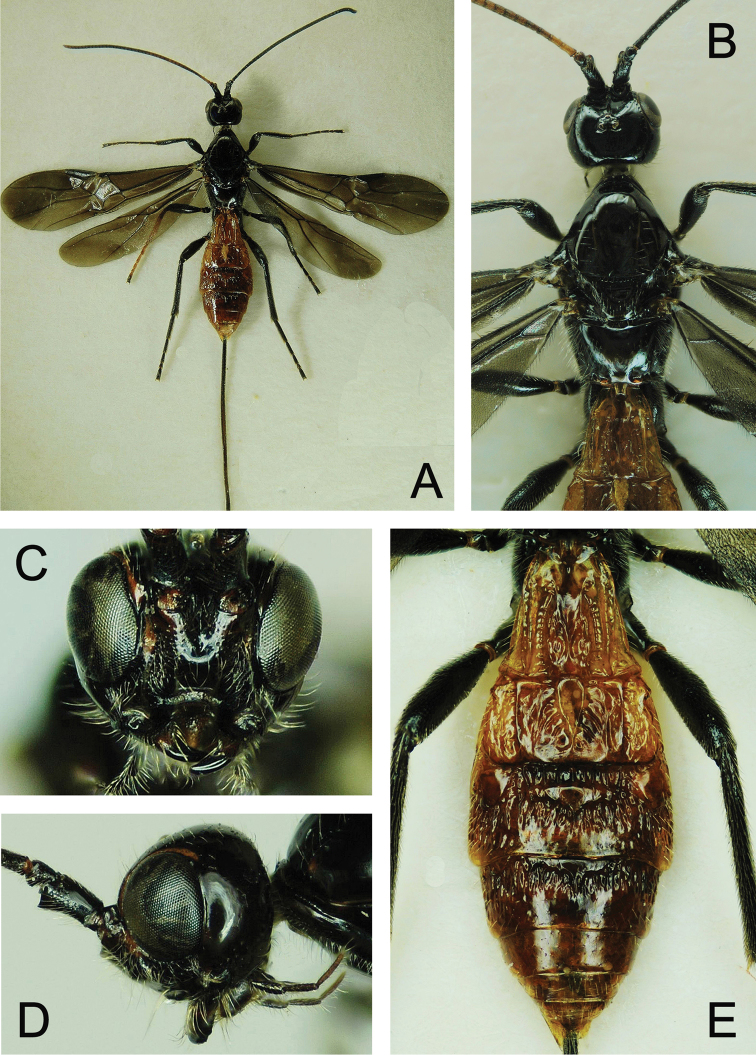
*Atanycolus ivanowi* ♀ **A** Habitus, dorsal view **B** head and mesosoma, dorsal view **C** head, frontal view **D** head, lateral view **E** metasoma, dorsal view.

**Figure 5.**
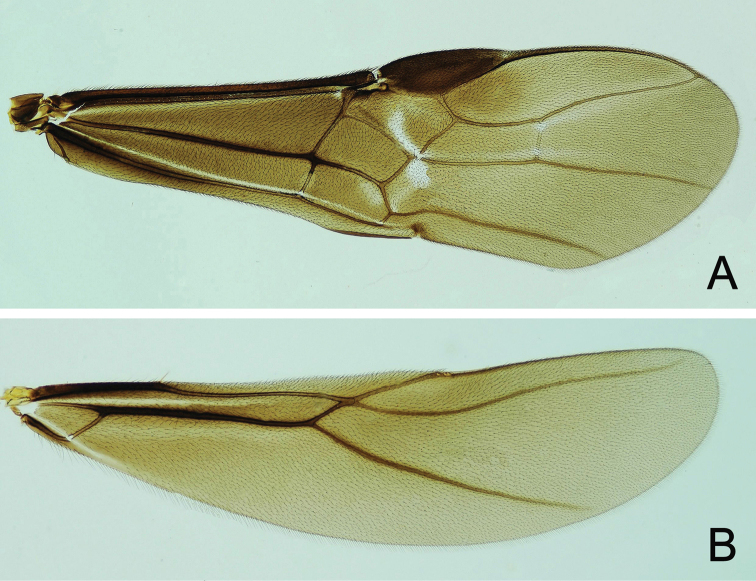
*Atanycolus ivanowi* ♀ **A** Forewing **B** Hind wing.

Figures 3A, B , 4 , 5

Vipio (Atanycolus) ivanowi Kokujev, 1898: 364.
Bracon (Vipio) sculpturatus Thomson, 1892: 1800; Wang et al., 2009. Syn. by Belokobylskij et al., 2003: 369.
Atanycolus signatus Szépligeti,1901, 33: 176. Syn. by Papp, 1960.

##### Material examined.

4♀, 20♂, China, Xinjiang Province, Gongliu City, Mohuer County, 25.VI.2006, 1325 m altitude, 43°15'27"N, 82°47'56"E, Yang ZhongQi leg. The collected cocoons were adhered to mature dead larvae of *A. mali*. 86♀, 78♂, same locality and biological data, but collected 25.VI.–24.VII.2015 by Wang ZhiYong.

##### Hosts.

Larva of *Agrilus mali* (**new record**) (Buprestidae). *Arhopalus syriacus* Reitter, *Leptura rubra* Linnaeus, *Monochamus galloprovincialis* Olivier *Osphranteria inaurata* Holzschuh, *Tetropium fuscum* Fabricius, *T. gabrieli* Weise (Cerambycidae); *Anthaxia aurulenta* Fabricius, *Chrysobothris solieri* Gory et Laporte, *Lampra mirifica* Mulsant, *Melanophila decastigma* Fabricius, *M. picta* Pallas, *Sphenoptera tappesi* Marseul (Buprestidae).

##### Distribution.

China (Xinjiang); Armenia; Austria; Azerbaijan; Croatia; Czech Republic; Finland; France; Germany; Greece; Hungary; Italy; Japan; Kazakhstan; Russia; Slovakia; Switzerland; Tajikistan; Turkey; Turkmenistan; Ukraine; Uzbekistan.

##### Remarks.

*Atanycolus ivanowi* is clearly characterised by the sculpture of the first to fourth metasomal tergites (Fig. 4E). Wang et al. (2009) reported *A. ivanowi* (as *A. sculpturatus*) for the first time from Xinjiang and also provided a key to Chinese fauna of *Atanycolus* Foerster.

Genus *Atanycolus* are ectoparasitoids of wood borers, and are usually solitary (Figure 3A), very rarely there are two individuals on one host. *A. ivanowi* is widely distributed in the Central and South Palaearctic region. This parasitoid is mostly found on the mature larva of *A. mali*. When full grown, the larva will spin a cocoon in the gallery of *A. mali*, ca. 10 days later the adult will emerge by biting a small round hole in the bark.

We found two generations per year of *A. ivanowi* in our experimental fields. The first generation lasts ca. 40 days from late March to May, and many adults can be seen during May to July. The larvae of the overwintering generation can be seen before August and later only cocoons can be found. Obviously, this parasitoid overwinters in the cocoon stage.

In total, we collected 86 females and 78 males of *A. ivanowi* in 2015 from one site (Xinjiang Province, Gongliu City, Mohuer County), which shows that the approximate ratio of female and male is 11/10. The natural parasitisation rate is approximately 26.7% and according to our investigations, *A. ivanowi* has the maximum population on *A. mali*. Obviously, *A. ivanowi* should be protected in order to increase the biodiversity of the forests, in which *Malus sieversii* is the main component.

### Doryctinae

#### Doryctes undulatus

Taxon classificationAnimaliaHymenopteraBraconidae

(Ratzeburg, 1852)

8e138285-3798-510f-94fd-e762982626f8

**Figure 6.**
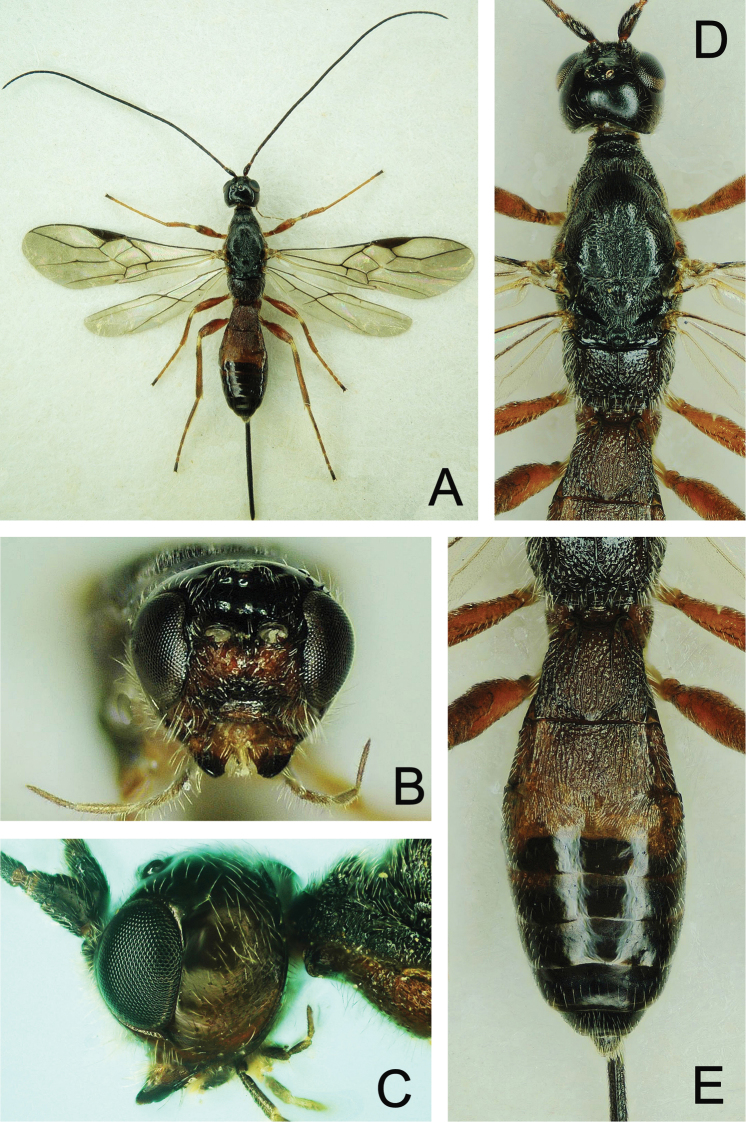
*Doryctes undulatus* ♀ **A** Habitus, dorsal view **B** Head, frontal view **C** Head, lateral view **D** Head and mesosoma, dorsal view **E** Metasoma, dorsal view.

**Figure 7.**
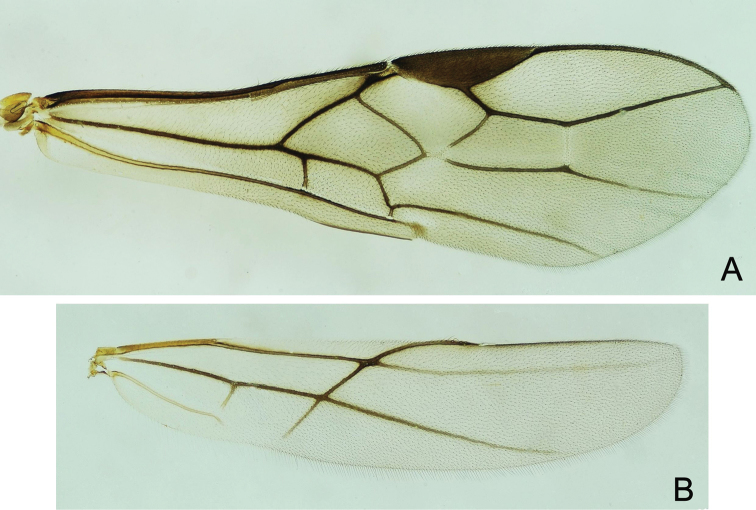
*Doryctes undulatus* ♀ **A** Forewing **B** Hind wing.

**Figure 8.**
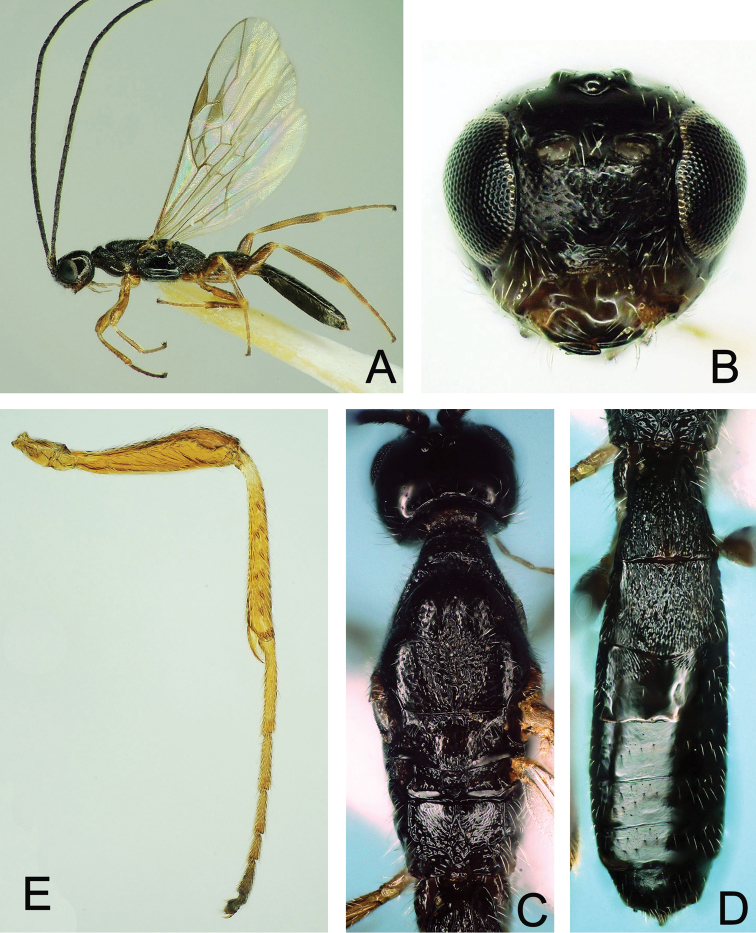
*Doryctes undulatus* ♂ **A** Habitus, lateral view **B** Head, frontal view **C** Head and mesosoma, dorsal view **D** Metasoma, dorsal view **E** Left fore leg.

Figures 3C, D, E, F , 6 , 7 , 8

Bracon undulatus Rarzeburg, 1852: 35.
Doryctes undulatus : Reinhard, 1865: 256; Marshall, 1888: 237; Shenefelt et Marsh, 1976: 1294; Belokobylskij, 1998: 63; Belokobylskij et Maeto, 2009: 128; Belokobylskij et al., 2012: 47.
Doryctes brachyurus : Marshall, 1888: 238; Shenefelt et Marsh, 1976: 1279; Papp, 1984: 175.

##### Material examined.

2♀, China, Xinjiang Province, Gongliu City, Mohuer County, 8.VIII.2008, 1325 m altitude, 43°13'25"N, 82°45'16"E, Yang ZhongQi leg. 1♀, China, Xinjiang Province, Xinyuan County, Halabula, 16.VI.2011, 46°12'16"N, 82°59'20"E, Yang ZhongQi leg., 25.VI.2011, hatched out from a mature larvae of *A. mali*. 3♀, China, Xinjiang Province, Xinyuan County, Halabula, 16.VI.2011, 46°12'16"N, 82°59'20"E, Yang ZhongQi leg., 10.VII.2011, hatched out from a mature larvae of *A. mali*. 1♂, China, Xinjiang Province, Xinyuan County, Halabula, 16.VI.2011, 46°12'16"N, 82°59'20"E, Yang ZhongQi leg., 25.VII.2011, hatched out from a mature larvae of *A. mali*. 1♂, China, Xinjiang Province, Gongliu City, Mohuer County, 15.VI.2011, 1325 m altitude, 43°13'25"N, 82°45'16"E, Tang YanLong, Wang ZhiYong & Yang ZhongQi leg., 8.VII.2011, hatched out from a mature larvae of *A. mali*.

##### Hosts.

Larva of *Agrilus mali* (**new record**) (Buprestidae). *Axinopalpis gracilis* Krynicki, *Grammoptera ruficornis* Fabricius, *Molorchus kiesenwetteri* Mulsant et Rey, *M. umbellatarum* Schreber, *Pogonocherus decoratus* Germar, *P. fasciculatus* DeGeer, *P. hispidulus* Piller, *P. hispidus* (Linnaeus), *Tetrops praeustus* (Linnaeus) (Cerambycidae); *Agrilus convexicollis* Redtenbacher, *A. cuprescens* Menetries, *A. mendax* Mannerheim *A. viridis* (Linnaeus) *Anthaxia tuerki* Scopoli (Buprestidae); *Magdalis armigera* Geoffroy, *M. ruficornis* (Linnaeus), *Pityogenes bidentatus* (Herbst) (Curculionidae).

##### Distribution.

China (Xinjiang, Heilongjiang, Jilin, Gansu); Bulgaria; France; Germany; Hungary; Italy; Japan; Kazakhstan; Korea; Lithuania; Moldova; Mongolia; Poland; Russia; Slovakia; Sweden; Switzerland; United Kingdom.

##### Remarks.

According to the detailed redescription of Japanese specimens (Belokobylskij et Maeto, 2009) and the key to this genus for China (Belokobylskij et al. 2012), our specimens from Xinjiang are quite the same, although there are still some tiny differences present, e.g., hind femur much stronger, 2.8 times as long as the maximum width in lateral view (vs. hind femur 2.9–3.2 times longer than wide in Japan); female body colour is stable reddish brown and male body is totally black (vs. reddish brown to almost black in Japan); extend of sculpture of third tergite varies from 1/4 to 1/2 basally, but semi-circular striation is always present, which is the main character to separate it from *D. striatellus* (Belokobylskij et Maeto 2009). After extensive comparison, we consider the differences to be intra-specific variation of *D. undulatus*. *Agrilus mali* is newly reported as host of *D. undulatus*. On average, a host larva is parasitised by two larvae of *D. undulates* as ectoparasitoid (Figure 3C, D, E, F).

#### Pareucorystes varinervis

Taxon classificationAnimaliaHymenopteraBraconidae

Tobias, 1961 (new record for China)

d6283b4a-d3fa-5afa-80dd-e364e9caf108

Figures 9 , 10

Pareucorystes varinervis Tobias, 1961: 529.
Pareucorystes depressus Fischer, 1966: 323.

##### Material examined.

10♀, 1♂, China, Qinghai Province, Hualong County, 17.VI.2008, 36°05'42"N, 102°15'43"E, Yang ZhongQi leg.

##### Host.

Larva of *Agrilus mali* (**new record**) (Buprestidae). *Agrilus angustulus* Illiger, *A. auricollis* Kiesenwetter, *A. convexicollis* Redtenbacher, *A. laticornis* Illiger, *A. sulcicollis* Lacordaire, *A. viridis* (Linnaeus) (Buprestidae); *Tetrops praeustus* (Linnaeus) (Cerambycidae).

##### Distribution.

China (Qinghai); Azerbaijan; Bulgaria; Canary Islands; Czechoslovakia; France; Hungary; Italy; Kazakhstan; Russia; Slovakia; Ukraine.

##### Remarks.

This species is mainly specialised on *Agrilus* species; if the population density could be enlarged, it would be a potential biological control agent. The species is also a new to the Chinese fauna, and a new parasitoid of *A. mali*.

**Figure 9.**
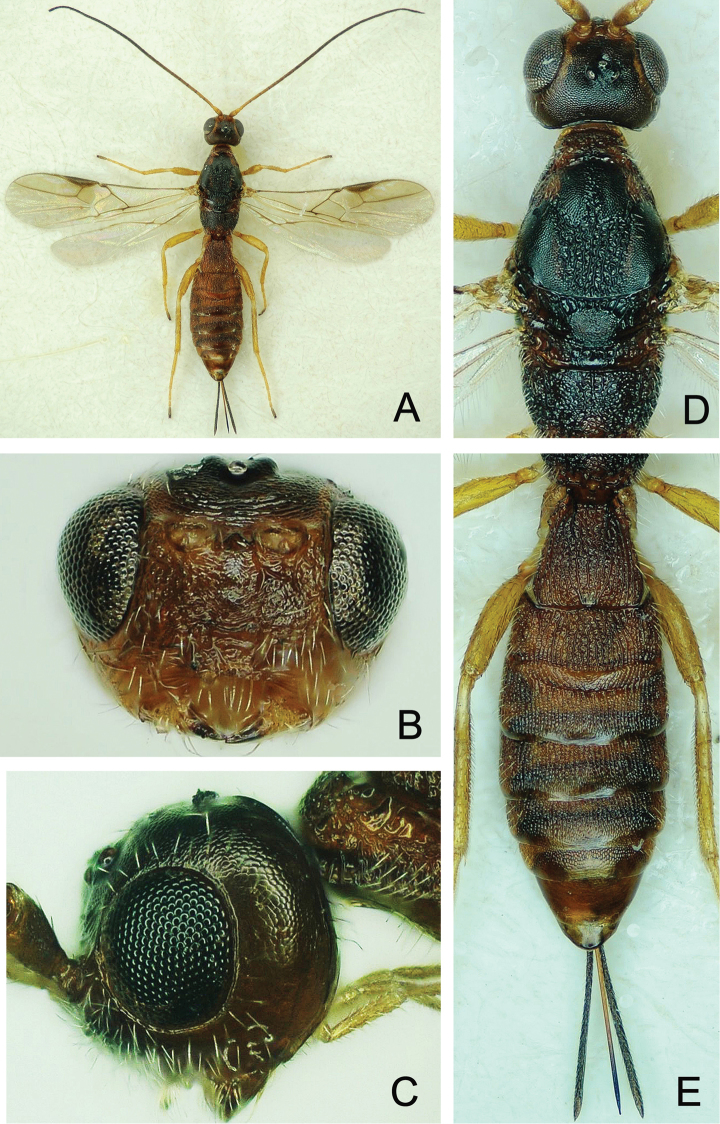
*Pareucorystes varinervis* ♀ **A** Habitus, dorsal view **B** Head, anterior view **C** Head, lateral view **D** Head and mesosoma, dorsal view **E** Metasoma, dorsal view.

**Figure 10.**
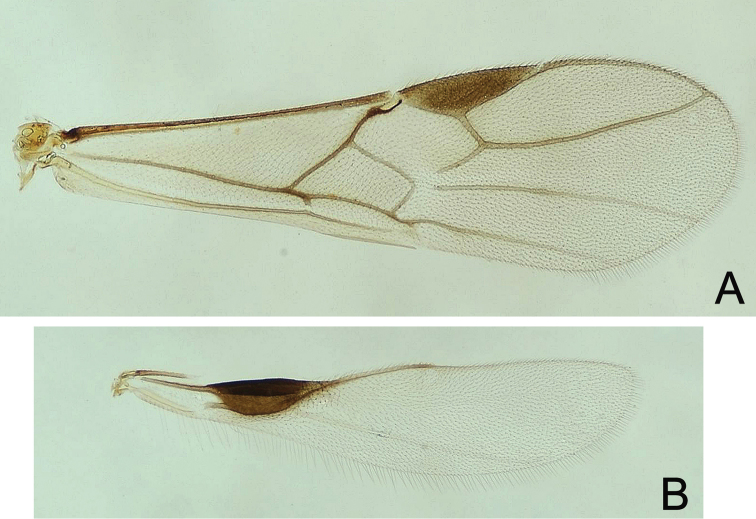
*Pareucorystes varinervis* ♂ **A** Forewing **B** Hind wing.

#### Polystenus rugosus

Taxon classificationAnimaliaHymenopteraBraconidae

Foerster, 1863

be3b17ae-fd02-5943-ad80-8338b1096187

Figures 11 , 12

Polystenus rugosus Foerster, 1863: 237; Shenefelt et Marsh, 1976: 1361; Papp, 1984: 182; Belokobylskij et Tobias, 1986: 34; Belokobylskij, 1998: 74; Belokobylskij et Maeto, 2009: 409; Tang et al., 2014: 6.
Corystes aciculatus Reinhard, 1865: 259.
Eucorystes aciculatus Marshall, 1888: 204.
Eucorystoides aciculatus Ashmead, 1900: 368; Shenefelt et Marsh, 1976: 1354; Papp, 1984: 182.

##### Material examined.

1♀, China, Shaanxi Province, Yijun County, 29.VII.2006, 35°23'56"N, 109°06'41"E, Yang ZhongQi leg, 28.VII.2006, hatched out from a mature larva of *A. mali*. 1♂, China, Xinjiang Province, Gongliu City, Mohuer County, 26.VI.2006, 1325 m altitude, 43°13'25"N, 82°45'16"E, Yang ZhongQi leg, 13.VII.2006, hatched out from a mature larvae of *A. mali*.

##### Hosts.

Larva of *Agrilus mali* (**new record**) (Buprestidae). *Agrilus angustulus* Illiger, *A. auricollis* Kiesenwetter, *A. sulcicollis* Lacordaire, *A. viridis* (Linnaeus), *Anthaxia manca* Linnaeus, *Coraebus bifasciatus* Olivier (Buprestidae); *Sinoxylon sexdentatum* Olivier (Bostrichidae).

##### Distribution.

China (Xinjiang, Shaanxi, Henan, Zhejiang, Taiwan?), Austria; Czech Republic; Germany; Hungary; Italy; Japan; Kazakhstan; Korea; Liechtenstein; Poland; Russia; Slovakia; Switzerland; Tajikistan; Ukraine.

##### Remarks.

This species is newly reported for Xinjiang and Shaanxi and *A. mali* is a new host record.

**Figure 11.**
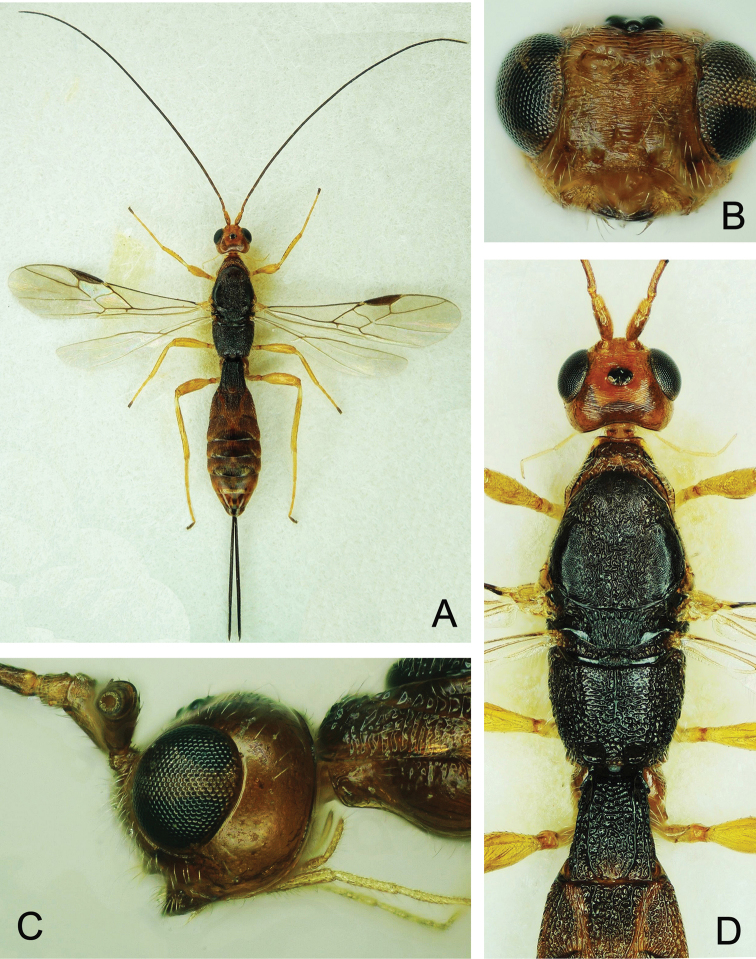
*Polystenus rugosus* ♀ **A** Habitus, dorsal view **B** Head, anterior view **C** Head, lateral view **D** Head and mesosoma, dorsal view.

**Figure 12.**
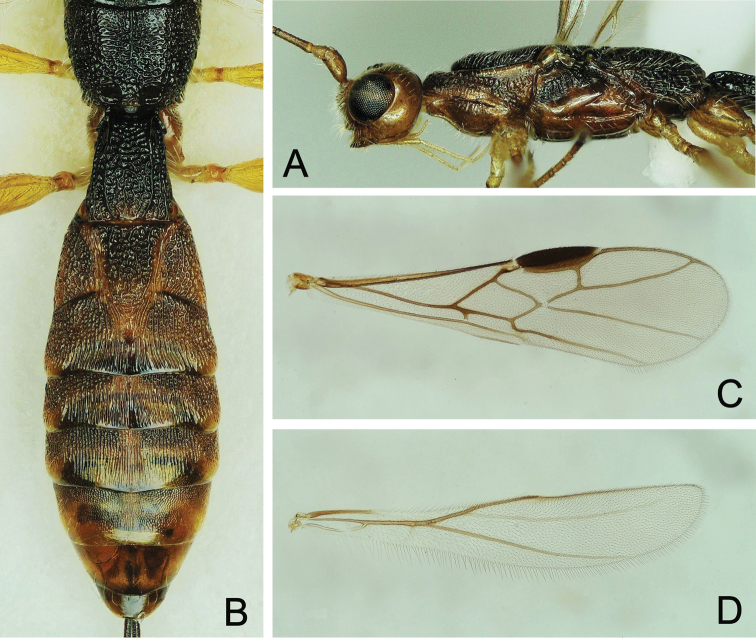
*Polystenus rugosus* ♀ **A** Head and mesosoma, lateral view **B** Metasoma, dorsal view **C** Forewing **D** Hind wing.

#### Spathius sinicus

Taxon classificationAnimaliaHymenopteraBraconidae

Chao, 1957

990f51a7-abb2-59df-aee0-1d40f22c65dc

Figures 13 , 14

Spathius sinicus Chao, 1957: 3; 1977: 209; Chen & Shi, 2004: 162; Tang et al., 2015: 106; Yu et al., 2016.

##### Material examined.

6♀, China, Xinjiang Province, Gongliu City, Mohuer County, 15.VI.2011, 1325 m altitude, 43°13'25"N, 82°45'16"E, Zhang YanLong, Wang ZhiYong & Yang ZhongQi leg, 8.VII.2011, hatched out from mature larvae of *A. mali*.

##### Host.

Larva of *A. mali* (**new record**) (Buprestidae).

##### Distribution.

China (Xinjiang, Fujian, Heilongjiang, Hunan, Shanghai, Jilin, Tianjin, Zhejiang); Japan.

##### Remarks.

This species is widely distributed in China and *A. mali* is the first reported of a host. It is peculiar that during several years of investigation, only 6 individuals have been found on *A. mali* at one tree, which indicates that it is an occasional parasitism.

**Figure 13.**
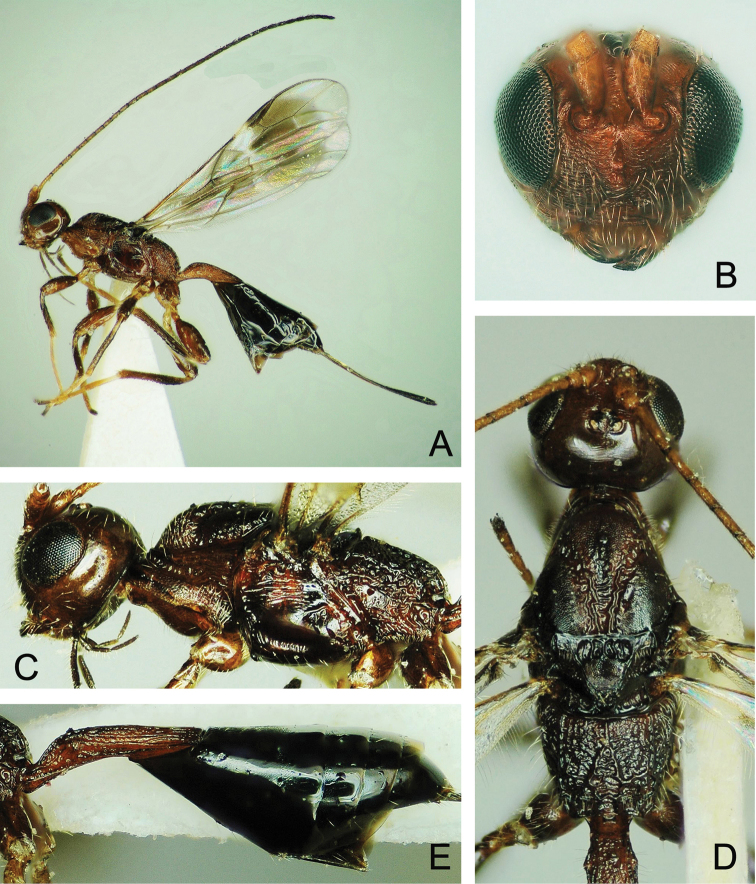
*Spathius sinicus* ♀ **A** Habitus, lateral view **B** Head, anterior view **C** Head and mesosoma, lateral view **D** Head and mesosoma, dorsal view **E** Metasoma, lateral view.

**Figure 14.**
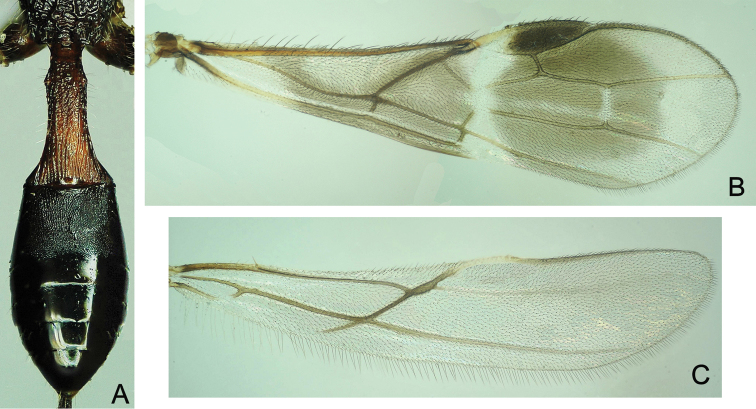
*Spathius sinicus* ♀ **A** Metasoma, dorsal view **B** Forewing **C** Hind wing.

#### Spathius brevicaudis

Taxon classificationAnimaliaHymenopteraBraconidae

Ratzeburg, 1844 (new record in China mainland)

55bc1e16-de72-5bb5-b437-72c6197d491e

**Figure 15.**
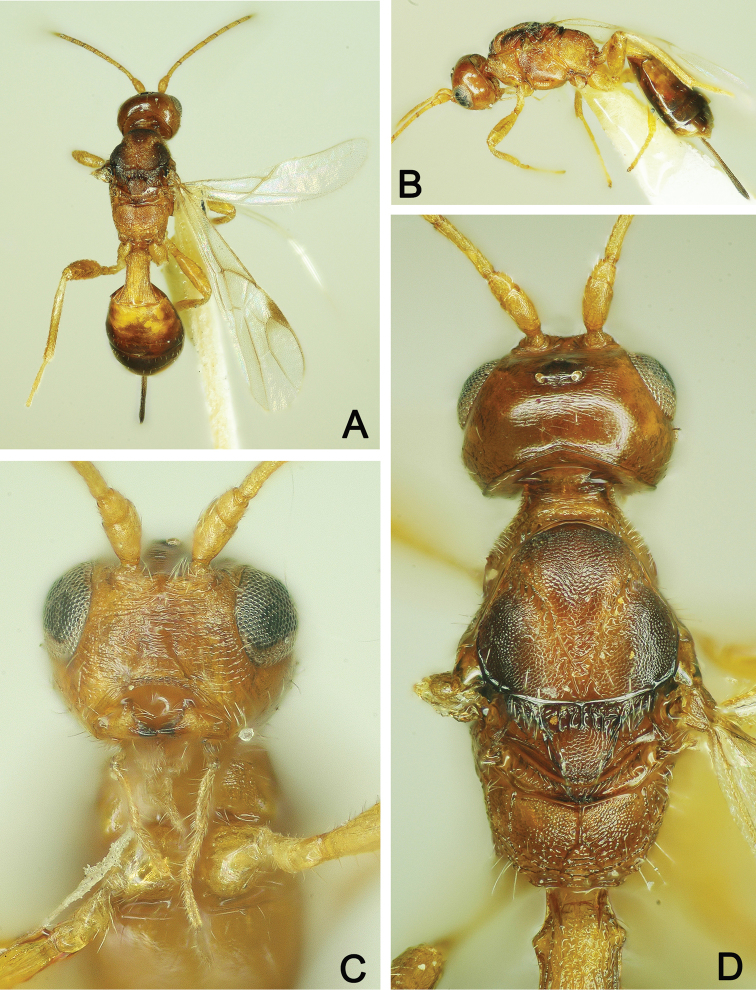
*Spathius brevicaudis* ♀ **A** Habitus, dorsal view **B** Habitus, lateral view **C** Head, anterior view **D** Head and mesosoma, dorsal view.

**Figure 16.**
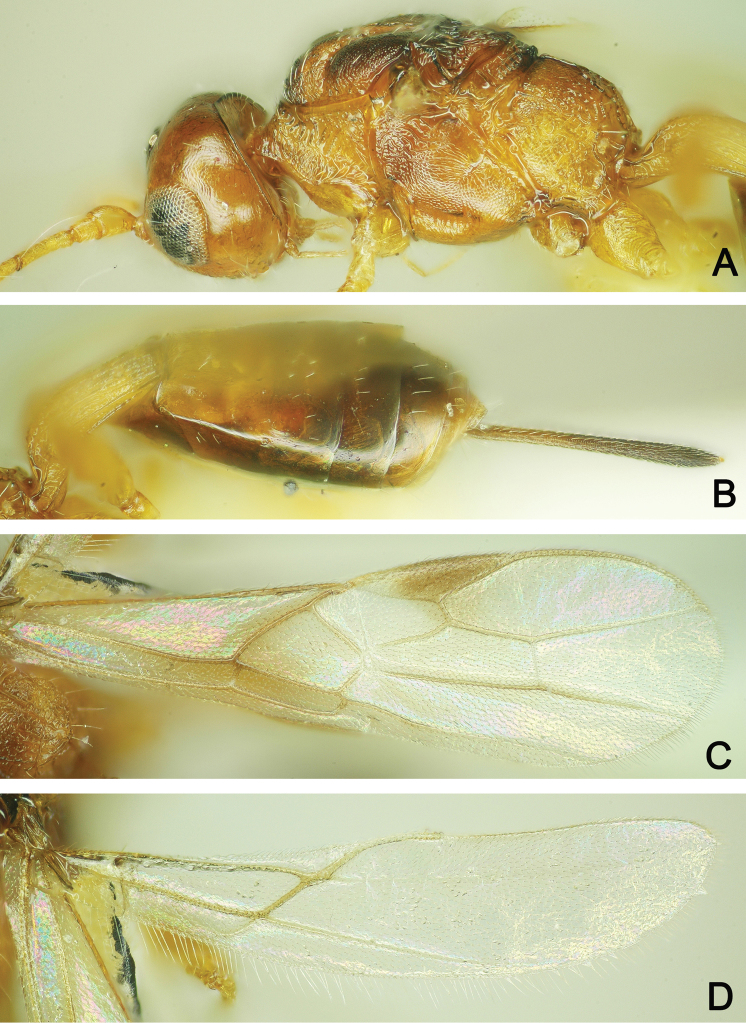
*Spathius brevicaudis* ♀ **A** Head and mesosoma, lateral view **B** Metasoma, lateral view **C** Fore wing **D** Hind wing.

**Figure 17.**
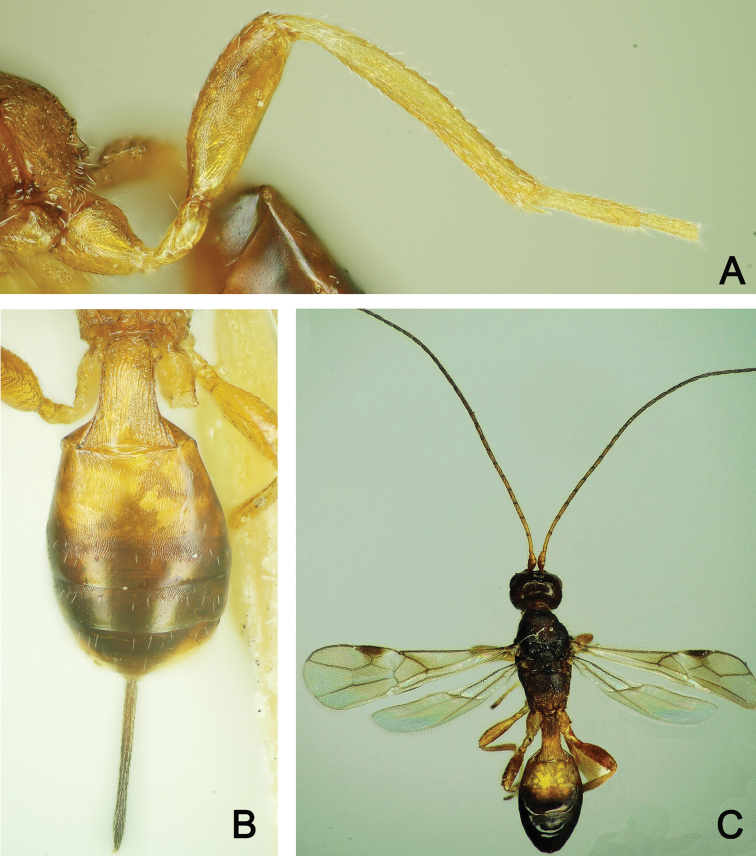
*Spathius brevicaudis***A** ♀, Left hind leg, lateral view **B** ♀, metasoma, dorsal view **C** ♂, habitus, dorsal view.

**Figure 18.**
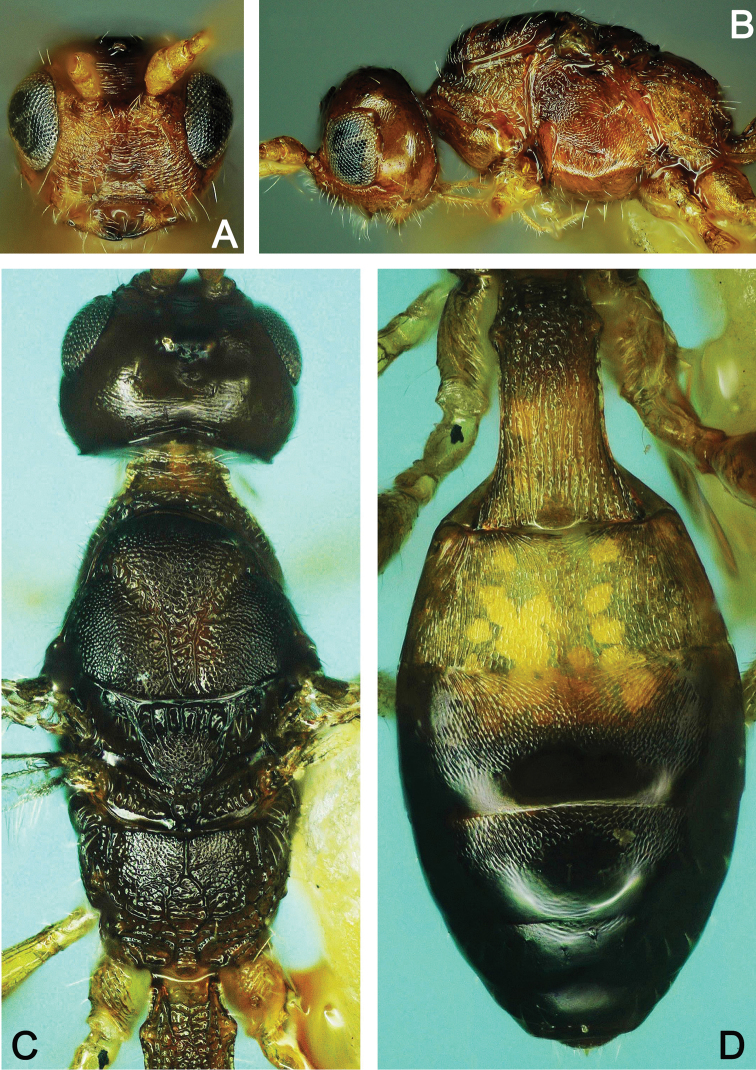
*Spathius brevicaudis* ♂ **A** Head, frontal view **B** Head and mesosoma, lateral view **C** Head and mesosoma, dorsal view **D** Metasoma, dorsal view.

Figures 15 , 16 , 17 , 18

Spathius brevicaudis Ratzeburg, 1844: 49; Nixon, 1943: 202; Belokobylskij, 1996: 188.

##### Material examined.

1♀, 1♂, China, Xinjiang Province, Gongliu City, Mohuer County, 26.VI.2006, 1325 m altitude, 43°13'25"N, 82°45'16"E, Zhang YanLong, Wang ZhiYong & Yang ZhongQi leg., 12.VII.2006, hatched out from mature larvae of *A. mali*.

##### Redescription.

Body length, ♀/♂ = 2.9/2.8 mm; forewing length, ♀/♂ = 2.34/2.25 mm.

##### Colour (Female).

Head dark brown, basal half of antenna yellow, its apical half brown; mesoscutal lobes, scutellum dark brown, pronotum brown; metasoma dark brown except first and second metasomal segments, basal portion of third metasomal segment yellow; fore wing partly weakly darkened; legs yellow (Fig. 15A, B).

##### Head.

Median length 0.7 times of its width in dorsal view; vertex broad, surface rough, with low (fine) sculptures and rare white setae (Fig. 15D); length between posterior margin of lateral ocellus and occipital carina half of head length in dorsal view; occipital carina median portion concave, reversed V-shaped (Fig. 15D); length of eye: length of temple in dorsal view = 4.3: 4; eyes small, slightly protruding bilaterally; OOL: OD: POL = 3: 1: 1.8; ocellar area distinctly differentiated, slightly swollen; width of head 1.1 times of height in front view, distance between eyes 1.3 × height of eye (Fig. 15C); face distinctly and irregularly striate, covered with white setae; malar space 0.6 × height of eye; height of clypeus 0.4 × its width, exterior margin of clypeus straight; basally mandible broad, apical portion black, blunt and robust; hypoclypeal depression deeply concave; antenna 28 segmented, scape twice length of first flagellar segment, and twice its maximum width; first flagellar segment 5.3× its maximum width, as long as second flagellar segment; last antennal segment acute apically.

##### Mesosoma.

Length of mesosoma 1.9 × its width and 1.6 × its height in lateral view (Fig. 16A); pronotal depression with short carinae; mesoscutum distinctly elevated above pronotum (Figs 15D, 16A). Mesoscutum nearly equilateral triangular, median length 0.9 × its maximum width; median and lateral lobes of mesoscutum with scaly sculpture; notauli and centre of mesoscutum deep and light-coloured with strong carinae (Fig. 15D); mesopleuron distinctly striate in upper 1/3 near pronotum and tegula, posterior 2/3 with scaly sculpture, epicnemial carina bent, episternal area taproot-shaped (Fig. 16A). Precoxal sulcus broad, length 1.67 × its width, with four longitudinal carinae inside. Scutellum flat and triangular, apical 1/3 of scutellum with scaly sculpture; scutellar sulcus 1/3 of scutellum length, with seven longitudinal carinae and separated small concave depressions; metanotum broad, dorsally concave, laterally with several longitudinal carinae, posterior margin slightly curved; propodeum weakly oblique posteriorly (lateral view), with scaly sculpture, medio-longitudinal carina bifurcates at basal third, posterior half of propodeum with irregular carinae.

##### Legs.

Fore femur 0.8 times as long as fore tibia and 3.75 times as long as its width, fore tibia 8.0 times of its width, outside with a row of spines and apex with comb of spines, ratio of fore tarsal segments I–V = 1.4:0.7:0.5:0.3:0.6; mid femur 0.8 times of mid tibia, ratio of mid tarsal segments I–V = 7:5:4:5:7; hind femur 2.7 times of its width, 0.8 times as long as hind tibia, ratio of hind tarsal segments I–V =1.5:0.8:0.5:0.4:0.8 (Fig. 17A).

##### Wings.

Fore wing with faintly brown along veins in basal half, apical half of fore wing largely subhyaline, its length 3.2 times of width; pterostigma 4.0 times as long as its maximum width; vein 1-R1 1.3 times of pterostigma, vein r originates at middle of pterostigma; vein SR1 8.5 times as long as vein r and straight; vein r nearly 1/4 of vein 2-SR, vein cu-a perpendicular to vein CU1, vein m-cu enters second submarginal cell; meeting point of veins 2-SR, 2-M and 2-SR+M finely sclerotised, veins reduced; vein 1-SR+M straight, vein 1-SR 1/3 length of vein 1-M; vein r-m weakly sclerotised, nearly invisible; veins 3-M and CU1a extending to wing margin (Fig. 16C). Length of hind wing 5.0 × its width (Fig. 16D).

##### Metasoma.

First tergite length 1.55 × its maximum apical width in dorsal view, apical 2/3 with regular longitudinal striae, basal 1/3 rugulose; in lateral view first tergite very robust, spiracular tubercles located at basal 1/4, laterally with erect white long setae, apical 1/3 of laterotergites visible; second tergite largely rugulose and with several large round yellow spots; basal 2/3 of third tergite striate-rugulose, apical 1/3 smooth; fourth tergite basally 1/4 with longitudinal rugulosity; fifth and sixth tergites smooth. Length of setose part of the ovipositor sheath 0.7 × length of metasoma, 0.38 × length of fore wing, and 0.3 × length of body (Figs 16B, 17B).

##### Male.

Body length 2.8 mm, otherwise similar to female (Figs 17C, 18), but pigmentation and metasomal sculpture more developed than in female.

##### Distribution.

China (Xinjiang, Taiwan); Austria; Azerbaijan; Bulgaria; Czech Republic; Denmark; France; Georgia; Germany; Hungary; Italy; Japan; Kazakhstan; Korea; Moldova; Mongolia; Poland; Romania; Russia; Slovakia; Sweden; Switzerland.

##### Host.

Larva of *Agrilus mali* (**new record**), *Agrilus viridis* (Linnaeus), *Anthaxia manca* Linnaeus, *A. quadripunctata* (Linnaeus), *Bostrichus bidens* Fabricius (Buprestidae); *Acanthocinus griseus* (Fabricius), *Arhopalus coreanus* Sharp, *Exocentrus lusitanus* (Linnaeus) (Cerambycidae); *Blastophagus minor* (Hartig), *B. piniperda* (Linnaeus), *Carphoborus minimus* (Fabricius), *Ceutorhynchus quadridens* (Panzer), *Dryocoetes autographus* (Ratzeburg), *Hylesinus fraxini* Panzer, *Ips acuminatus* (Gyllenhal), *I. typographus* (Linnaeus), *Lixus bidens* Fabricius, *Magdalis frontalis* Gyllenhal, *M. violacea* (Linnaeus), *Niphades variegatus* Roelofs, *Onthotomicus angulatus* Eichhoff, *Phloeotribus rhododactylus* (Marsham), *Pissodes notatus* Fabricius, *P. obscurus* Roelofs, *Pityogenes bidentatus* (Herbst), *P. chalcographus* (Linnaeus), *Pityophthorus micrographus* (Linnaeus), *Polygraphus subopacus* Thomson, *Rynchaenus fagi* (Linnaeus), *R. pilosus* Fabricius, *R. quercus* (Linnaeus) *R. salicis* (Linnaeus), *R. testaceus* Muller, *Scolytus intricatus* (Ratzeburg), *S. koenigi* Schewyrew, *S. laevis* Chapuis, *S. mali* (Bechstein), *S. multistriatus* (Marsham), *S. rugulosus* (Muller), *Shirahoshizo insidiosus* Roelefs, *Sh. pini* Morimoto, *Sh. rufescens* Roelofs (Curculionoidea); *Xiphydria longicollis* (Geoffroy) (Xiphydriidae).

##### Remarks.

Among all the parasitoids of *A. mali* we found in the past years, only two specimens of *S. brevicaudis* were recorded, which indicates that it is an occasional parasitoid of this host. This species is here recorded as new for continental China, after Belokobylskij (1996) reported it from Taiwan. Its identification is based on Ratzeburg’s original description, Nixon’s redescription, and reared material from Europe seen by the second author. *S. brevicaudis* may be confused with *S. rubidus* (Rossi), but *S. brevicaudis* has vein M+CU1 of fore wing straight or nearly so (weakly to moderately sinuate in *S. rubidis*), wing membrane with a faintly infuscate patch or band below pterostigma (with a distinct dark patch or band below pterostigma, rarely reduced in small specimens) and basal pale spot of pterostigma weakly differentiated (basal pale spot of pterostigma distinctly differentiated in dark specimens). Although, the differences are minor, all related to the fore wing and sometimes gradual, we prefer to recognise *S. brevicaudis* as separate species till molecular data will become available. The main reason for this is that reared series show these minor differences to be stable enough for separation both species in north-western Europe.

### Key to braconid parasitoids of *Agrilus mali* in northwest China

**Table:** 

1	Occipital and prepectal carinae absent (Fig. 4D)	***Atanycolus ivanowi* (Kokujev)**
–	Occipital and prepectal carinae present (Figs 6C, 8C, 9C, 11C, 13C, 15D)	**2**
2	Forewing with two submarginal cells, because vein r-m of fore wing is completely absent (except sometimes in *Pareucorystes*) (Figs 9A, 10A, 12C)	**3**
–	Forewing with three submarginal cells, because vein r-m is weakly developed (Figs 7A, 14B, 16C)	**4**
3	Metasomal tergites 2+3 with V-shaped pale area and without posteriorly curved transverse groove (Fig. 12B)	***Polystenus rugosus* Foerster**
–	Metasomal tergites 2+3 without V-shaped pale area and with posteriorly curved transverse groove (Fig. 9E)	***Pareucorystes varinervis* Tobias**
4	Vein m-cu of fore wing entering first submarginal cell (antefurcal: Fig. 7A); first metasomal tergite sessile (Fig. 6E)	***Doryctes undulatus* (Ratzeburg)**
–	Vein m-cu of fore wing entering second submarginal cell (postfurcal: Figs 14B, 16C); first tergite petiolate (Figs 14A, 17B)	**5**
5	Forewing partly strongly infuscated (Fig. 14B); first metasomal tergite elongate and slender, approx. twice as long as its apical width (Fig. 14A)	***Spathius sinicus* Chao**
–	Forewing weakly infuscated, subhyaline (Fig. 16C); first tergite comparatively short and broad, approx. 1.5 × as long as its apical width (Fig. 17B)	***Spathius brevicaudis* Ratzeburg**

## Supplementary Material

XML Treatment for Atanycolus ivanowi

XML Treatment for Doryctes undulatus

XML Treatment for Pareucorystes varinervis

XML Treatment for Polystenus rugosus

XML Treatment for Spathius sinicus

XML Treatment for Spathius brevicaudis
